# Comparative reproductive ecology of Old and New World Trogons, an order in decline across the world

**DOI:** 10.1002/ece3.11273

**Published:** 2024-04-10

**Authors:** Necmiye Şahin Arslan, Thomas E. Martin

**Affiliations:** ^1^ Montana Cooperative Wildlife Research Unit University of Montana Missoula Montana USA; ^2^ Alaca Avni Çelik Vocational School Hitit University Corum Turkey

**Keywords:** Borneo, life history traits, nestling growth, parental behavior, tropics, Venezuela, wing development

## Abstract

Many tropical species show declining populations. The pantropical order Trogoniformes has 76% of its species ranked as declining, reflecting a worldwide problem. Here, we report on the reproductive ecology and life history traits of the declining and near‐threatened old world Whitehead's Trogon (*Harpactes whiteheadi*), the declining new world Collared Trogon (*Trogon collaris*), and the stable Masked Trogon (*T. personatus*). We also reviewed the literature on reproductive ecology and life history traits of trogons to assess possible commonalities that might help explain population declines. We found that the declining Whitehead's and Collared Trogons had reasonable nest success (32% and 25%, respectively), while the stable Masked Trogon had poor reproductive success (9%), all contrary to population trends. However, the limited literature data suggested that poor reproductive success may be common among trogons, which may contribute to population declines. Parents fed young at a low rate and had long on‐bouts for incubation and nestling warming that reduced activity at the nest, as favored by high nest predation risk over evolutionary time. We found that young fledged from the nest with poorly developed wings, as also favored by high nest predation risk. Evolved nestling periods among trogon species suggests that poor wing development is likely common. Wing development has been shown to affect juvenile survival after leaving the nest. The poor wing development may be an important contributor to population declines that deserves more attention. Evolved life history traits are important to recognize as creating population vulnerabilities in a changing world.

## Graphical abstract

We examine the avian order Trogoniformes, which has 76% of its species classified as declining and assess possible reasons for such worldwide declines. We provide field data on breeding biology and life history for three species and review the literature for the order. We provide possible reasons for population declines based on nesting success and a novel argument based on developmental state at fledging that affects juvenile survival.

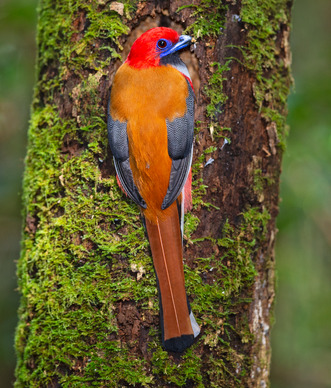

## 1 INTRODUCTION

Many tropical species (Ceballos et al., 2015; Gibson et al., 2011), but the demographic causes of the population declines remain poorly studied for most species.

One taxonomic group that has shown broad population declines across the world is the avian order Trogoniformes; 31 of 41 trogon species ranked by IUCN were classified as having declining populations (Table 1). Trogons can be an important component of tropical ecosystems. Many trogon species excavate nest holes in tree trunks and arboreal termite nests (Brightsmith, 2004; Skutch, 1942, 1948, 1962). These holes can provide habitats for a variety of other species and reflect an ecosystem engineer function of trogons (Valdivia‐Hoeflich et al., 2005). Understanding possible causes of their population declines, therefore, is of great importance.

**TABLE 1:** Population trends and IUCN conservation status in Trogoniformes.

Region	Genera	Species	Increasing/stable	Decreasing	IUCN conversation status	
					Vulnerable	Near‐threatened
New World	4	26	6	20	0	2
Africa	1	3	2	1	0	0
Asia	2	12	2	10	1	6

*Note*: Two New World species were not included by IUCN (2023).

The order Trogoniformes includes a single family, Trogonidae, that includes 43 species in seven genera (Gill et al., 2023). Of these, three species are in Africa in a single genus. Twelve species in two genera are in Asia. Six species are Neotropical quetzals in two genera and the remaining 22 species are Neotropical trogons in two genera (Gill et al., 2023; Johnsgard, 2000). According to the IUCN (2023), declining population trends are typical of most of the family members across all of these regions (Table 1). The reason for such broad declines among trogons remains unclear. As we will summarize in this article, trogon reproduction and life history traits have had very little detailed study.

Detailed reproductive ecology and life history studies of trogons are important from two standpoints. First, they can shed light on possible causes of their population declines and aid future conservation. Second, evolved life history strategies can affect population responses to environmental perturbations and susceptibility to extinction (Clark & Martin, 2007; Martin & Mouton, 2020; Webb et al., 2002). Moreover, refinements of life history theory depend on study of life history traits across diverse species that differ in evolved life history strategies (Martin, 1996, 2004, 2015). Importantly, evolved life history strategies of hole‐nesting birds generally differ from other nest types, even in the tropics (Cody, 1966; Martin, 1995) and, thus, adding important diversification of life history strategies. Yet, detailed studies of the reproductive ecology of hole‐nesting species in the tropics are uncommon, and especially for trogons. Moreover, comparisons of Old World and Neotropical species are lacking, yet both regions show population declines and have near‐threatened species (Table 1). Here, we provide detailed comparative field data on one old world (Asian) trogon studied in Malaysian Borneo (Whitehead's, *Harpactes whiteheadi*) and two Neotropical (Collared *Trogon collaris* and Masked *T. personatus*) trogons studied in Venezuela.

Whitehead's Trogon is one of eight trogon species that are considered as near‐threatened by the IUCN (IUCN, 2023). No information is available on reproductive and life history ecology of this species, and not even adult mass has been reported (Collar, 2020). Given its near‐threatened status and the total lack of information on its demography, understanding its reproductive and life history ecology is particularly important. This need is further emphasized by a study of the reproductive ecology of two declining congeneric species (Orange‐breasted *H. oreskios* and Red‐headed *H. erythrocephalus*) in a large national park in Thailand (Steward & Pierce, 2011). These two congeners had extremely low reproductive success (Steward & Pierce, 2011), which may indicate an important influence of reproduction for their declining populations. Yet, data from more species are needed to better understand the generality of such possibilities. Our study of the declining and near‐threatened congeneric Whitehead's Trogon thereby provides an important comparison, while also providing the first details of its life history.

Comparisons of reproductive ecology between declining and stable species can also add insight into the possible causes of population declines. The two Neotropical trogons studied here are considered Least Concern by the IUCN (2023) but include one that is decreasing (Collared Trogon) and one with a stable population (Masked Trogon). The latter thereby, provides a nice contrast with Whitehead's Trogon from the standpoint of population trends. The Whitehead's Trogon genus *Harpactes* is distantly related to the Neotropical genus *Trogon* (De Los Monteros, 1998; Moyle, 2005; Oliveros et al., 2020). Yet, they are ecologically similar in their dependence on nesting in holes and, thereby, provide an important contrast.

Here we report details on the breeding ecology and life history for the near‐threatened Old World Whitehead's Trogon in Borneo and two Neotropical trogon species, Collared and Masked Trogons. We also present a literature summary of the life history data for Trogoniformes.

## 2 METHODS

Whitehead's Trogon was studied in Kinabalu Park, Sabah, Malaysian Borneo (6°05′ N, 116°33′ E), a 754 km^2^ protected area of primary forest. Research was conducted during the 2009–2020 breeding seasons from early February to mid‐June. Seven study plots were established at elevations of 1450–1950 m. These plots were contiguously located and included ca. 560 ha, with each plot ca. 60–70 ha in size (Martin & Mouton, 2020).

Collared and Masked Trogons were studied in the northern Andes in Yacambú National Park, a 269 km^2^ area in Lara State, western Venezuela (9°38′ N 69°40′ W). The fieldwork was restricted to primary cloud forest habitat between 1400 and 2000 m, encompassing a similar elevation range to our study in Borneo. Data were collected during seven breeding seasons from 2002 to 2008 and from late February to early July. Research was conducted on seven study plots similar in size (ca. 60–70 ha) to those on the Borneo site (Martin & Mouton, 2020). These trogons were not focal study species, such that we did not collect as comprehensive data as for the Whitehead's Trogon.

In general, the same standardized data collection methods were used in both Borneo and Venezuela studies, described as following. We located nests by observational cues of breeding pairs and systematic search (Martin & Geupel, 1993; Şahin Arslan et al., 2023; Şahin Arslan & Martin, 2019), and measured the nest and nest‐substrate heights using clinometers. We obtained the elevation of the nest location with a GPS device (Garmin, Olathe, Kansas, USA) for Whitehead's Trogon.

A nest initiation date was specified as the day the first egg was laid in a nest, and the egg‐laying season was characterized by the distribution of nest initiation dates. Nests were checked daily during egg‐laying and the first 2 days of incubation to obtain the exact day the last egg was laid to ascertain the start day of incubation. If a nest was found during incubation and was of unknown age, we checked the nest daily until hatch. Nests were also checked daily or twice daily near hatching and fledging to obtain exact timing of transitions for measuring incubation and nestling period lengths (Martin, Oteyza, Boyce, et al., 2015; Martin, Oteyza, Mitchell, et al., 2015; Şahin Arslan et al., 2023). Otherwise, nests were generally checked every other day in Borneo, but from 1 to 4 days in Venezuela, to determine status and predation (Martin & Geupel, 1993). Clutch size was only used from nests located during building or egg‐laying. We did not include nests observed later to ensure no partial loss was included (Martin et al., 2006). The incubation period was defined as the number of days between the last egg laid and last egg hatched (Martin et al., 2007; Nice, 1954). The nestling period was defined as the days between the last egg hatched and the last nestling fledged and only used for nests where the last egg laid and hatch days were observed within 24 h of precision (Martin, Lloyd, et al., 2011).

Daily nest predation rates and daily survival rates were estimated using maximum likelihood estimation via the Mayfield method (Hensler & Nichols, 1981; Mayfield, 1961, 1975). This method is highly correlated with the logistic exposure method (Şahin Arslan & Martin, 2024; Shaffer, 2004) but allows more ready comparisons with the wider availability of Mayfield estimates in the literature. We considered a nest successful if parents were observed feeding young outside the nest or the young left within 2 days of normal fledging age. If nest contents disappeared earlier, we considered it to be due to predation.

We used an electronic scale with 0.001 g accuracy (ACCULAB, Elk Grove, Illinois, USA) to weigh fresh eggs on the day the last egg was laid or within the first 2 days of incubation. Nestlings were weighed for the first 3 days and then every other day throughout the rest of the nestling period, while also measuring wing chord and tarsus length using calipers (Mitutoyo) with an accuracy of 0.01 mm. As a part of a banding program, some adults were captured using mist‐nets, and their mass, wing chord, and tarsus lengths were measured.

Parental behavior at nests was recorded using video cameras for Whitehead's Trogon during both incubation and nestling stages starting near sunrise. We put 30× zoom video‐cameras 4–10 m from the nests and camouflaged the cameras to prevent possible disturbance. We generally sought 6‐h video recordings of parental behavior at a nest, but they varied from 4 to 9 h each day of video recording (mean duration during incubation = 5.96 ± 0.24 h, *N =* 27; during nestling period = 6.33 ± 0.13 h, *N =* 97). Parental activity of the two trogon species in Venezuela were not video‐recorded. Video recordings were used to quantify incubation nest attentiveness, as well as brooding attentiveness and feeding rates during the nestling period (Martin, Oteyza, Boyce, et al., 2015; Martin, Oteyza, Mitchell, et al., 2015; Şahin Arslan & Martin, 2019). Incubation nest attentiveness was measured as the percent of total video time that a parent sat on the eggs for each day of video recording (Martin, Oteyza, Boyce, et al., 2015). Brooding attentiveness for nestlings was calculated as the percent of video time that a parent sat on the nestlings for each day of video‐recording, and feeding rates as the number of feeding trips of both parents to the nest^−h^ for that recording.

### 2.1 Statistics

We conducted all analyses in R.4.2.2 (R Core Team, 2022) and we present mean values with standard errors, ranges, and sample sizes. We estimated growth rate constants (K) for mass, tarsus length, and wing chord using the logistic growth model (Remeš & Martin, 2002). The model is based on the equation: *W*(*t*) = *A*/1 + e (−K*(*t* − *t*_*i*_)), where *W*(*t*) is body mass, tarsus length, or wing chord length, *A* is the asymptotic size, *t* is age and *t*_*i*_ is the age at the inflection point where growth rate changes from accelerating to decelerating, and *K* is the maximum rate of growth which is obtained at the inflection point (Martin, 2015). We tested for differences in the growth curves between Whitehead's and Collared Trogons using the nls function in R, and using nest identity as a random effect, while specifying the above equation and running a model for each species and then testing for model differences between species using anova.

We used generalized linear mixed‐effects models through the glmer function in the *lme4* package (Bates et al., 2015) to investigate the fixed effect of nestling age and brood size on feeding rate, with nest identity as a random effect. Brooding behavior changed in a backward logistic curve (Şahin Arslan et al., 2023) and is described by the same three parameters as for growth rate above, where in this case, *A* = asymptote at hatching day, *K* = instantaneous rate of change at the inflection time point, and *t* = the inflection time point where the curve changes from accelerating to decelerating. We used the SSlogis function in the *nlme* package (Pinheiro, Bates, & R Core Team, 2023) to describe the relationship and test for differences between brood sizes in slope (*K*), intercept (*A*), and inflection time point (*t*) of brooding behavior by Whitehead's Trogon while using nest identity as a random effect. *p* ≤ .05 was considered as statistically significant throughout.

## 3 RESULTS

**FIGURE 1:**
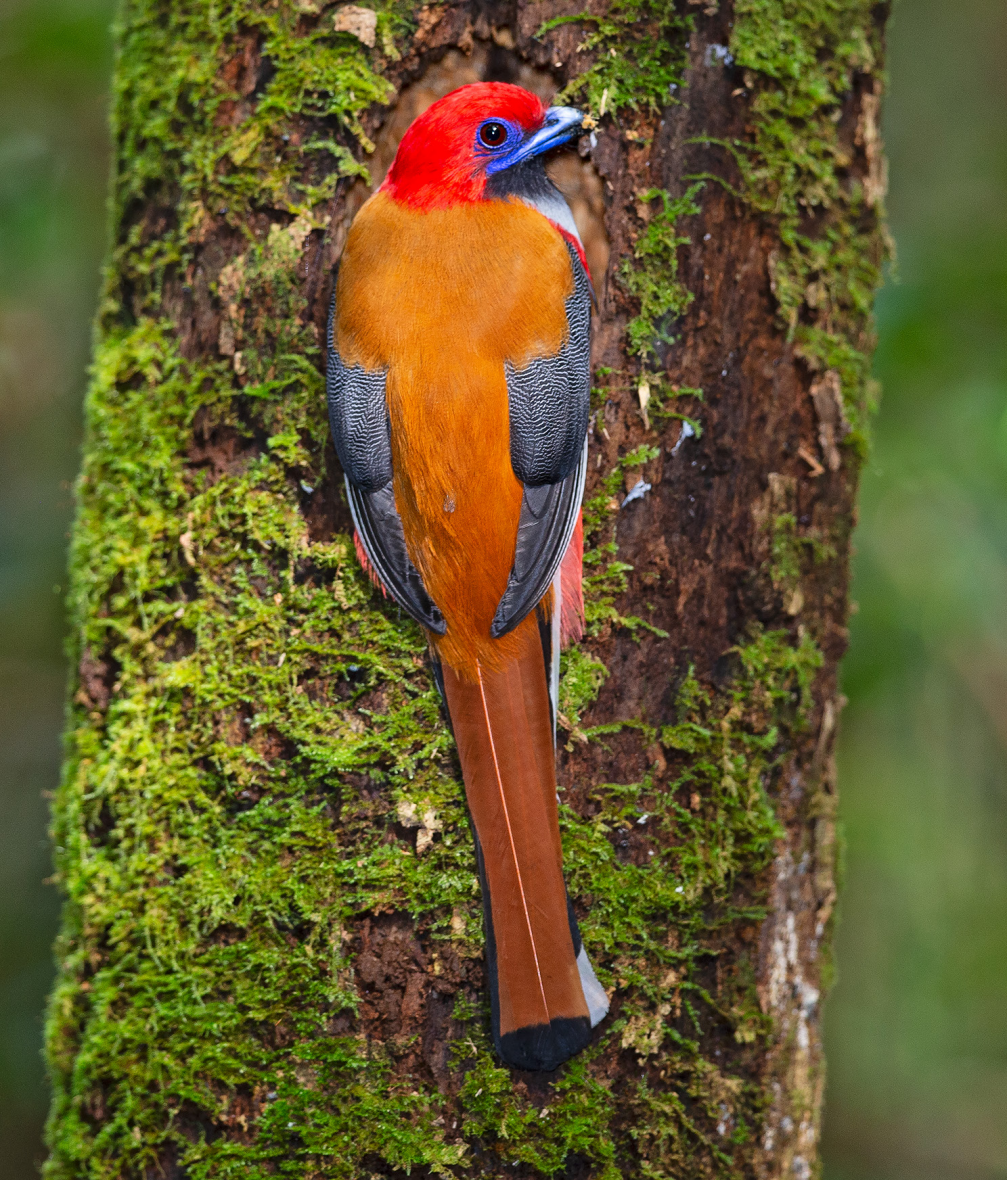
Whitehead's Trogon excavating a nest hole in Malaysian Borneo. Note the rotten wood in the tip of its bill. Photo by T. E. Martin.

We located 38, 20, and 16 nests of Whitehead's, Collared and Masked Trogons, respectively. Whitehead's excavated its nest at an average height of 4.4 ± 0.31 m (*n* = 38, range: 1.75–9.0 m) on 5.7 ± 0.53 m height trees (*n* = 38, range: 2.3–20 m) (Figure 1). They excavated their nests in dead trees (snags) for 80% of the nests, with 11% in live trees, and the remainder in old stumps. Average and median elevations of nests were 1597 and 1592.5 m, respectively (range: 1472–1866). Nest and nest‐substrate height were only measured at two nests of Collared Trogon and they were 1.9 and 2.0 m from the ground in a 3.5 stump and 17 m snag, respectively. For Masked Trogon, nest height from the ground was 2.5 ± 0.5 m (*n* = 3, range: 1.5–3.0) and the one nest substrate height measured was a 2.0 m stump. Both Collared and Masked Trogons had 58% and 44%, respectively, of their nests in snags, and 21% and 38%, respectively in old stumps, with the remainder in live trees.

Adult mass of Whitehead's Trogon was 84.4 ± 1.24 g (*n* = 15, range: 78–94 g), and tarsus and wing chord lengths were 15.9 ± 0.33 mm (*n* = 14, range: 13.9–17.8 mm) and 134.6 ± 0.96 mm (*n* = 16, range: 127–140 mm). An adult Collared Trogon was not captured, so morphological data were not obtained. One adult male Masked Trogon was captured and weighed 68.0 g, with a wing chord of 126.0 mm and tarsus length of 16.8 mm.

All three trogons skipped 1 day between laying eggs. Based on the nests we located in building and laying stages, all three species laid one or two eggs, and had similar mean clutch sizes (*F* = 2.24, *p* = .12, Table 2). Although we only observed nests with one or two eggs, we found one Whitehead's nest with three nestlings (Table 2). Egg mass varied from 10.6% to 12.4% of adult mass across the three trogon species, and clutch mass averaged slightly more than 20% for all three species (Table 2).

**TABLE 2:** Mean ± 1SE, range, and (sample size) of clutch size, early incubation egg mass, and adult mass for the three trogons studied here, plus the percentage of adult mass represented by egg and clutch (mean egg mass × mean clutch size) mass.

	Trogon species		
	Whitehead's	Collared	Masked
Clutch size (eggs)	1.96 ± 0.04 1–2 [3]^a^ (28)	2.0 ± 0.0 2 (18)	1.83 ± 0.11 1–2 (12)
Egg mass (g)	8.93 ± 0.15 7.0–10.04 (25)	7.11 ± 0.13 5.98–8.16 (21)	8.41 ± 0.11 7.66–8.90 (10)
Adult mass (g)	84.4 ± 1.24 78–94 (15)	64.4^b^ – (47)	68.0 – (1)
% Egg/adult mass	10.6	11.0	12.4
% Clutch/adult mass	20.7	22.1	22.6

^a^ One nest was found with three nestlings, which is not included in the mean clutch size.

^b^ Hartman (1961) (from Panama).

**FIGURE 2:**
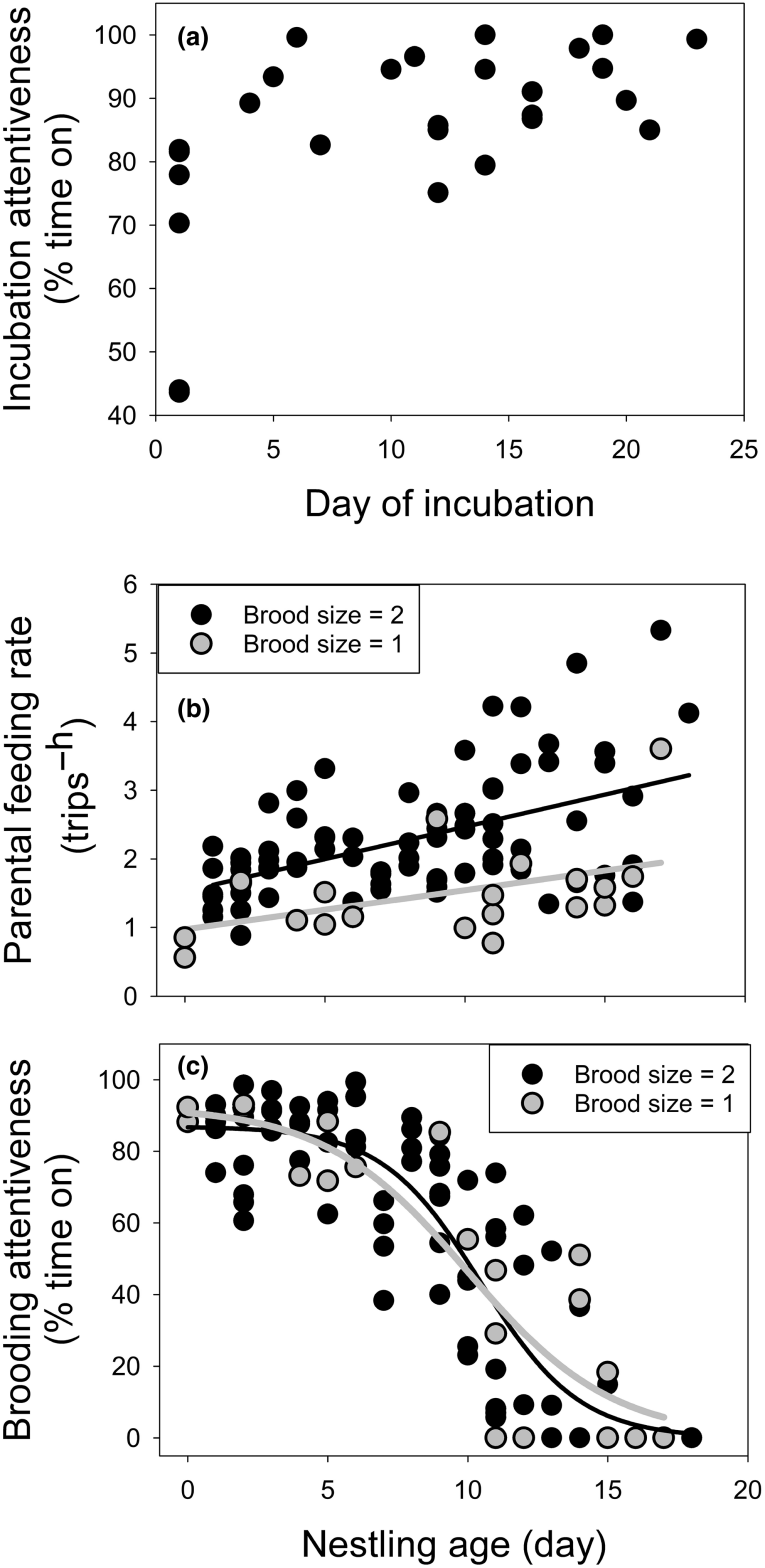
(a) Incubation attentiveness, (b) brooding attentiveness, and (c) feeding rates across egg and nestling ages in Whitehead's Trogon in Malaysian Borneo. Day 0 during incubation is the day the last egg was laid. Day 0 for the nestling period is hatch day. For brooding and feeding rates, closed circles reflect brood sizes of two nestlings, and open circles represent brood sizes of one nestling. *A* = Asymptote, effectively estimated near hatch day, *t*_*i*_ = timing (day) of the maximum slope or inflection point where the slope switches from accelerating to decelerating, *K* = the maximum rate of decrease in brooding, estimated at the inflection point.

Incubation and nestling periods were 23.1 ± 0.51 days (*n* = 5 nests, range: 22–24.5 days) and 17.0 ± 0.36 days (*n* = 9, range: 15.5–18.0 days), respectively in Whitehead's Trogon. Incubation period for Collared was 19 ± 0.0 days (*n* = 2 nests) and was not obtained for Masked Trogon. Both parents incubated, brooded, and fed the nestlings in all three species. Incubation attentiveness in Whitehead's Trogon showed large variation on the first day of incubation (the day after the last egg laid) with a range of 43.6%–81.9% (mean = 66.6 ± 7.39%) among six nests (Figure 2a). Attentiveness did not change across the rest of the incubation period (from day 4 on) (*t* = 0.92, *p* = .36, *N =* 16 nests, 161 video hours, mean per nest of 6.0 ± 0.24 h) and averaged 90.8 ± 1.56% (Figure 2a). On‐bout duration could not be estimated because most bouts were sufficiently long that recording ended before bouts did, reflecting their high attentiveness. Attentiveness was not measured in the two Venezuelan trogons.

The rate that Whitehead's Trogon visited the nest to feed nestlings increased with nestling age (*t* = 1.020, *p* < .001) and brood size (*t* = 2.469, *p* < .001) with similar slopes between brood sizes (age × brood size: *t* = 0.315, *p* = .753, *N =* 23 nests, 614 video hours, mean per nest = 6.3 ± 0.13 h) (Figure 2b). The logistic curve parameters for brooding behavior (Figure 2c) did not differ between brood sizes for the asymptote or inflection time (*A*: *t* = 0.01, *p =* .99; *t*: *t* = 0.25, *p =* .81). However, slopes differed (*K*: *t* = −2.13, *p =* .04) with the slope for brood size of one young being slightly less steep than for two young, although sample size was not strong for singletons (Figure 2c). The estimated asymptote (i.e., hatching day) from the logistic curve of both brood sizes combined for time spent brooding nestlings by parents was 86.9 ± 3.36%. Brooding attentiveness remained similar through day 6 and then decreased rapidly (*K* = −0.566 ± 0.097), with the inflection point at 10.5 ± 0.30 days (Figure 2c). Parental behavior during the nestling period was not measured for the two Venezuelan trogon species.

**FIGURE 3:**
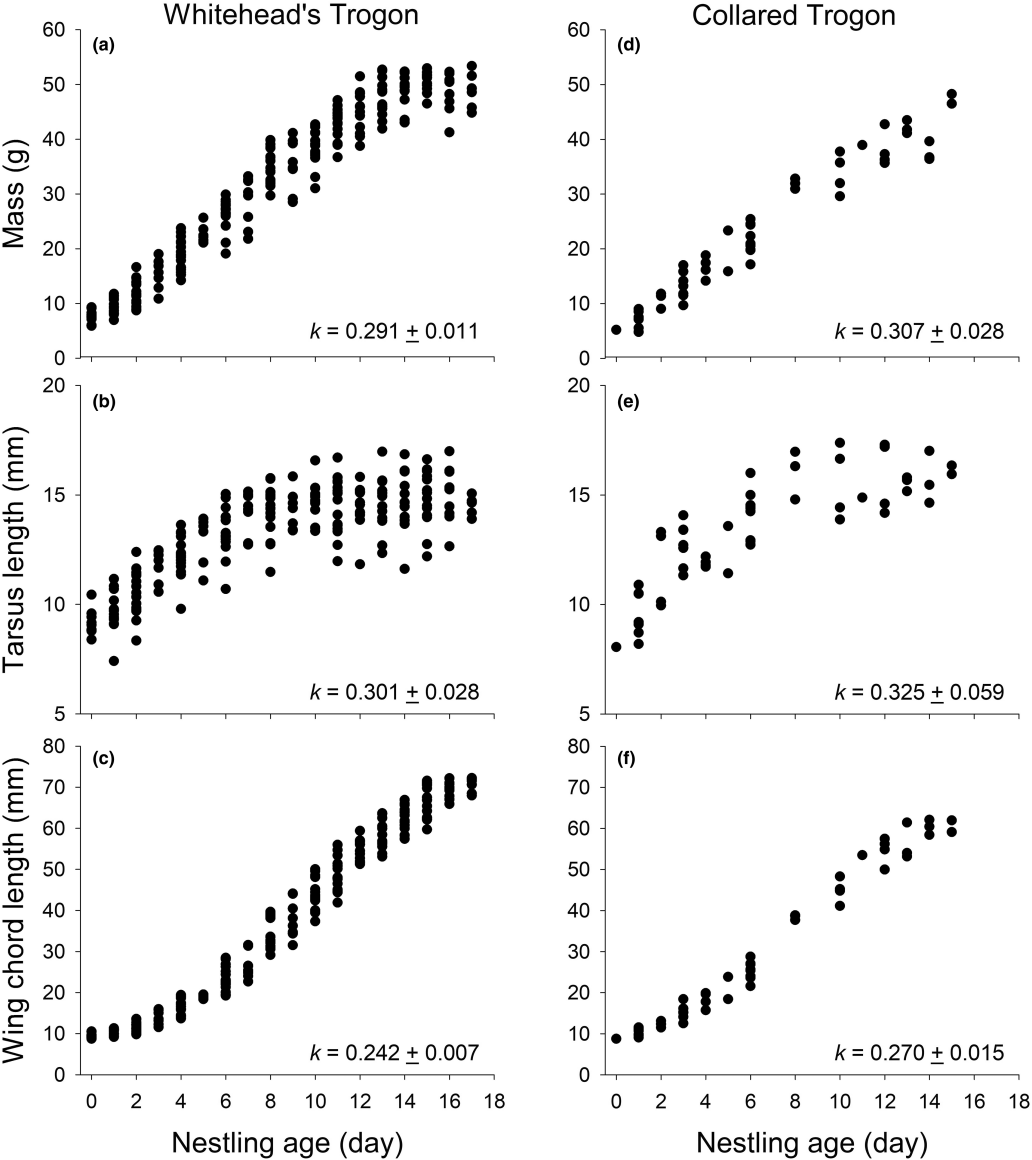
Growth curves of nestling Whitehead's and Collared Trogons in Malaysian Borneo and Venezuela, respectively, based on (a, d) mass, (b, e) tarsus length, and (c, f) wing chord. *K* = Growth rate constant. Sample sizes = 19 nests, with 222, 223, and 223 measurements of mass, wing length, and tarsus length, respectively for Whitehead's Trogon and nine nests with 53, 54, and 54 measurements, respectively, for Collared Trogon.

Growth rate constants (*K*) for Whitehead's Trogon nestlings were 0.291, 0.301, and 0.242 for mass, tarsus length, and wing chord length, respectively (Figure 3a–c). Growth rate constants (*K*) were somewhat similar for Collared Trogon nestlings, although a little higher (Figure 3d–f). Tests of the growth equations indicated they did not differ between species for mass, tarsus, or wing chord lengths (mass: *F* = 1.33, *p* = .12; tarsus: *F* = 0.45, *p* = .99; wing chord: *F* = 0.83, *p* = .80). However, we were not able to measure nestlings through fledging in Collared Trogons, and the shortened measurement period reduces asymptotic change near fledging and can artificially increase the estimates. Ultimately, the growth curves look quite similar through day 15 for both Whitehead's and Collared Trogons (Figure 3). We were only able to measure nestlings in one nest of Masked Trogons and for only part of the period, eliminating the ability to estimate anything about their growth rates.

One of the most surprising elements of nestling growth was that they fledged at 51.8 ± 0.38% (*N* = 15 fledglings) of adult wing size in Whitehead's Trogon (Figure 3c vs. Table 2). We could not estimate this for Collared Trogons because we were not able to measure fledglings on fledge day, but the similar growth curves (Figure 3c vs. Figure 3f) and nestling periods suggest a similar proportional wing size at fledging. Such small wing development can affect flight ability (Martin, 2014; Martin, Tobalske, et al., 2018). Mass of nestling Whitehead's Trogon on fledge day also was small relative to adult size (Figure 3a vs. Table 2) at 57.6 ± 1.01% (*N* = 15). However, fledglings almost achieved adult tarsus size, reaching 93.12 ± 1.72% of adult size on fledge day (Figure 3b vs. Table 2).

Initiation dates (first egg laid in a nest) lasted from February 25 to June 3 with a median date of March 26 for Whitehead's Trogon (*n =* 36 nests) in Malaysian Borneo. Collared (April 25–June 13, *n* = 10 nests) and Masked Trogons (April 3–June 14, *n* = 9 nests) had shorter nesting seasons in Venezuela. Although the small sample sizes raise question about actual season lengths.

Overall daily nest survival rates varied from 0.974 to 0.937 (Table 3) among the three trogons species, but did not differ statistically (*χ* ^2^ = 3.21, *p =* .20). Masked Trogon, with the smallest sample size, had the lowest nest success, with Whitehead's and Collared Trogon exhibiting similar rates (Table 3). Overall daily nest predation rates varied from 0.025 to 0.046 among the three trogon species (Table 3) with Masked Trogons again having the highest rate. The estimated percentage of nests that were successful (daily survival rate^nesting cycle length^) was 32% for the typical 43‐day nesting cycle of Whitehead's Trogon. Collared and Masked Trogons are similar in size to the congeneric Mountain and Elegant Trogons (Table 4) and their incubation period (19 days each) was same as we found for the Collared Trogon (Table 4). If we assume a similar nestling period of 15 days for the Collared and Masked Trogons and 3 days for egg‐laying of a typical clutch of two eggs, it yields an approximate nesting cycle length of 37 days. Using this estimate, the percentage of successful nests for Collared and Masked Trogons is 25% and 9%, respectively.

**TABLE 3:** Daily predation and survival rates with ± standard errors in the three trogon species.

	Trogon species		
	Whitehead's	Collared	Masked
Daily predation rate in incubation period	0.034 ± 0.010 (319.5)	0.018 ± 0.010 (165.5)	Not enough data
Daily predation rate in nestling period	0.013 ± 0.006 (314)	0.054 ± 0.026 (73.5)	Not enough data
Overall daily predation rate	0.025 ± 0.006 (651.5)	0.028 ± 0.011 (246)	0.047 ± 0.019 (127.5)
Overall daily survival rate	0.974 ± 0.006 (651.5)	0.963 ± 0.012 (246)	0.937 ± 0.021 (127.5)

*Note*: Numbers of exposure days are represented in parentheses for 38, 20, and 16 nests of the Whitehead's, Collared, and Masked Trogons, respectively.

**TABLE 4:** Summary of life history and breeding data from the literature on Trogoniformes species.

Species	Adult mass (g)	Egg mass (g)	Clutch size (# eggs)	Incubation period (day)	Nestling period (day)	Feeding rate (trips/nstl^=h^)	Nest height (m)	Latitude	Reference
Narina Trogon (*Apaloderma narina*)	67.8		2.6* 2–3^+^	16–17* 18–20^+^	25, 28			S. Africa*, Zambia^+^, Kenya^+^	1
Orange‐breasted (*Harpactes oreskios*)	57.3		2.4 ± 0.1 (17)	17.5 ± 0.5 (2)	12–14+	1.1 ± 0.2 (2)	2.1 ± 0.2 (19)	14°26′ N	2
Red‐headed (*H. erythrocephalus*)	80.3		2.6 ± 0.1 (48)	18 ± 0.2 (9)	13.4 ± 0.4 (6)	1 ± 0.1 (2)	2 ± 0.1 (49)	14°26′ N	2
Whitehead's (*H. whiteheadi*)	84.4		1.96 ± 0.04 (28)	23.1 ± 0.51 (5)	17 ± 0.4 (9)	1.37 ± 0.11 (15)	4.4 ± 0.31 (38)	6°05′ N	3
Eared Quetzal (*Euptilotis neoxenus*)	122.5		2.8 ± 0.9 (28)^4^	22 (1)^4^	29–31 (5)^4^		11.4 ± 4.1 (14)^4^ 13.1, 17.8^5^	28°28 to 30°33′ N	4 5
Resplendent Quetzal (*Pharomachrus mocinno*)	202.5	17 ± 2.2 (4)^6^	2^6^, 2^7^ 2.0 (3)^8^ 2.0 (3)^9^	18–19 (2)^6^ 17–18 (1)^7^	29 (2)^7^	1.39^6^	8.8 ± 3.5 (43)^6^ 10.8 ± 1.86 (11)^9^	10°3′ N^6^ 9°4′ N^7^ 14°6′ N^8,9^	6,7, 8,9
White‐tailed Quetzal (*Pharomachrus fulgidus*)	160	16.0 (2)	2 (1)				2.45, 4.0	11°06′ N	10
Pavonine Quetzal (*Pharomachrus pavoninus*)	176.7	8.5, 13.5	2 (1)		21–24 (1)		4.2 (1)	12°33′ S	11
Black‐throated Trogon (*T. rufus*)	53.8		2.0 ± 0 (7)	18 (1)	14–15	1.0	1.3–3.7 (8)	9°4′ N	12
Elegant Trogon (*T. elegans*)	70.9		2–4	19 ± 1.7 (5)	15 ± 3.1 (5)	1.0		31–32° N	13
Mountain Trogon (*T. mexicanus*)	71		2 (1)	19 (1)	15–16		0.83–1.2 (3)	14°45′ N	14
Masked (*T. personatus*)	63.4	8.41 ± 0.11 (10)	1.83 ± 0.11 (12)				2.5 ± 0.50 (3)	9°38′ N	3
Collared (*Trogon collaris*)	64.2	7.11 ± 0.13 (21)^3^	2.0 ± 0 (18)^3^ 2 (1) ^15^	19.0 ± 0 (2)^3^		0.53 (1)^15^	1.95 ± 0.05 (2)^3^ 3.7 (1)^15^	9°38′ N ^3^ 9°4′ N ^15^	3 15
Ecuadorean Trogon (*T. mesurus*)		11.0 ± 0.29 (3)	3.0 (2)					4°23′ S	16
Slaty‐tailed Trogon (*T. massena*)	141	14.1–15.8^17^	3 (3)^18^				2.6–5.6 (5)^18^	9°2′ N^18^ 15°4′ N^18^	17, 18
Black‐headed Trogon (*T. melanocephalus*)	85.1		3.0 (3)^19^	19 (1)^19^ 17–18 (2)^20^	16–17 (1)^19^ 16–17^20^		0.6–1.5 (3)^19^	15°26′ N^19^ 9°5′ N^20^	19, 20
Green‐backed Trogon (*T. viridis*)	89.7	10.5^22^	2.25 (4)^21^	16–17^21^	25^21^		2–5.5 (5)^21^	9°4′ N^21^	21, 22
Guianan Trogon (*T. violaceous*)	51.5		≥3 (1)		≥17		4.5–30	9°4′ N	23

*Note*: Sample sizes are shown in parenthesis. ^+,^*show data from different locations that were presented in the same reference. Body mass data is from Dunning Jr (2007). 1 Fry et al. (1988); 2 Steward and Pierce (2011); 3 This Study; 4 González‐Rojas et al. (2008); 5 Lammertink et al. (1996); 6 Wheelwright (1983); 7 Skutch (1944); 8 LaBastille et al. (1972); 9 Bowes and Allen (1969); 10 Pulgarin‐R and Laverde‐R (2015); 11 Lebbin (2007); 12 Skutch (1959); 13 Hall and Karublan (1996); 14 Skutch (1942); 15 Skutch (1956); 16 Schulenberg and Greeney (2020); 17 Johnsgard (2000); 18 Skutch (1972); 19 Skutch (1948); 20 Riehl (2005); 21 Skutch (1962); 22 Haverschmidt (1968); and 23 Skutch (1972).

## 4 DISCUSSION

Reproductive ecology and life history strategies can play an important role in differential population responses and extinction risk of species to environmental perturbations (Martin & Mouton, 2020; Webb et al., 2002). The large proportion of species in the order Trogoniformes that show declining populations across the world (76% of 41 species, Table 1) emphasizes the importance of studying their reproductive ecology and life histories as possible influences on their population trends. Moreover, they serve an important ecosystem function by creating habitat for other species (Valdivia‐Hoeflich et al., 2005).

We found low reproductive success (9%) in the Masked Trogon, which is ranked as having a stable population (IUCN, 2023). However, low reproductive success in trogons is not uncommon. Two species studied in Thailand (Orange‐breasted and Red‐headed Trogons) also had less than 9% of nests successfully producing young (Steward & Pierce, 2011). The Narina Trogon in Kenya was reported to produce four young in eight nests where clutch size is 2–3 eggs (Fry et al., 1988). If we assume a mean clutch size of 2.5, then these eight nests had 20 eggs and the four young represented a maximum success rate of 20%, although not taking into account the stage that nests were found and exposure days that would undoubtedly reduce the success rate (Hensler & Nichols, 1981; Mayfield, 1961). Skutch (1959, 1976) reported only one of seven nests (14%) was successful in the Black‐throated Trogon (*T. rufus*) in Costa Rica, although again not accounting for stage and exposure days. Wheelwright (1983) reported 67%–78% failure of nine nests, while Skutch (1944) and LaBastille et al. (1972) reported 100% failure of five nests in the Resplendent Quetzal (*Pharomachrus mocinno*). The low nest success seen among these trogons is interesting in that hole‐nesting birds generally have high nest success (Lack, 1948; Martin, 1995; Nice, 1957), but trogon nest holes are larger than for many other hole‐nesting birds. Ultimately, low reproductive success may be common among trogon species, although sample sizes are generally low and more detailed studies are needed in more locations.

An understanding of the impact of reproductive success on population trends depends on also knowing adult survival probabilities. Neither adult survival rates nor lifespan has been well‐studied for any species within the Trogoniformes (Winkler et al., 2020). Fogden (1972) reported four of four (100%) adult Scarlet‐rumped Trogons (Harpactes duvaucelii) survived for 1 year. We very rarely recaptured or resighted Whitehead's Trogons, but one female banded as an adult (i.e., at least 1 year old, but age unknown) was recaptured 3 and 6 years later. Although anecdotal, it may indicate long lifespans, and based on clutch sizes of two eggs, we would expect reasonably high adult survival as typical of other tropical bird species (Martin et al., 2017). The number of broods attempted per year is unclear, with single‐brooding reported for Elegant Trogon (*T. elegans*) (Hall & Karublan, 1996) and double‐brooding reported for Resplendant Quetzals (Wheelwright, 1983). Riehl (2005) reported that at least two pairs laid second clutches in the Black‐headed Trogon, but did not specify whether these followed successful or failed first nests. Single‐brooding might be common because trogons may care for their fledglings for as much 17 weeks after fledging (Fogden, 1972). Skutch (1942) claimed that he never observed double‐brooding among any trogon species other than Resplendant Quetzals. We never observed double‐brooding in Whitehead's Trogon and the relatively short duration of the nesting season suggested a single brood. Even if we assume 1.25 broods per year (occasional double‐brooding) and 90% annual adult survival probability, nest success below 15% with a clutch size of two would still produce declining populations (see Clark & Martin, 2007; Martin & Mouton, 2020). Thus, the low nesting success reported above for many trogon species could indicate that poor reproduction is an important contributor to the population declines broadly observed among trogons (Table 1). Yet, detailed studies of possible multibrooding and adult survival probability are needed.

On the other hand, low nest success is not true of all trogons or at least all locations. We found 32% nest success in Whitehead's Trogon (Table 3). González‐Rojas et al. (2008) found that 25 of 29 nests (86%) of the Eared Quetzal (*Euptilotis neoxenus*) produced at least one fledgling. Riehl (2005) found 10 of 14 nests (71%) were successful in Black‐headed Trogon. Certainly, reproductive success of the Eared Quetzal and Black‐headed Trogon was sufficient to maintain stable populations if juvenile survival is also reasonable (but see next paragraph), and the nest success of Whitehead's Trogon may also be sufficient for a stable population (see Martin & Mouton, 2020). Yet, all three species are ranked as declining (IUCN, 2023). Masked Trogon had the lowest nest success of our three species, but it was the only one of the three ranked as having a stable population (IUCN, 2023). Collared Trogon had reasonable nest success for maintaining populations (see Martin & Mouton, 2020) but is also ranked as decreasing (IUCN, 2023). Nest success based on small sample sizes from a single area may not match well with population trends. Moreover, nest success does not include possible problems created by poor availability and competition for nest sites thought to limit nesting in some cases (Brightsmith, 2005; González‐Rojas et al., 2008). Clearly, more detailed studies of reproduction across more locations are needed among trogons to understand its contribution to declining populations.

Another potentially important population limit in trogons may be juvenile survival. Parents fed at a low average rate (1.4 feeding trips per nestling^−h^) in Whitehead's Trogon (Figure 3) that was typical of other trogons (Hall & Karublan, 1996; Lammertink et al., 1996; Skutch, 1959, 1976; Steward & Pierce, 2011). This low feeding rate may contribute to the slow nestling growth (Figure 2). Moreover, we report here for the first time that wing development at fledging for Whitehead's Trogon is quite poor, and proportionally shorter than among any other species we have studied (Cheng & Martin, 2012; Martin, 2014; Martin, Tobalske, et al., 2018). Wing development affects flight performance and fledgling survival (Martin, Tobalske, et al., 2018). The poor wing development of Whitehead's Trogon is likely true of Collared Trogon (Figure 3) suggesting that wing development may be poor among many trogon species. Indeed, the duration of the nestling period for Whitehead's and Collared Trogons was typical of most trogons, other than quetzals (Table 4), further suggesting that poor wing development at fledging is likely typical of trogons. This fits with observations that poor flight ability at fledging has been observed in trogons (Johnsgard, 2000). Fledging at a poorer developmental stage can result from high nest predation rates over evolutionary time favoring earlier leaving of nests to reduce risk (Bosque & Bosque, 1995; Martin, 1995, 2014; Martin, Tobalske, et al., 2018).

The poor wing development at fledging documented here and implied by short nestling periods in many other trogon species (Table 4) may further suggest that relatively high nest predation may be typical for trogons over evolutionary time, and fledgling survival may be problematic in the current changing landscapes. Of course, not all trogons show such short nestling periods. The quetzals, in particular, show longer periods (Table 4). Yet, Winter (1998), in talking with biologists using radio‐tracking to study Resplendant Quetzals, reported that less than 20% of juveniles survived to breed, thereby suggesting that their longer nestling periods were still not sufficient for high juvenile survival. Such possibilities reveal the importance of considering evolved life history strategies for understanding demographic responses to environmental perturbations (also Martin & Mouton, 2020) and possible causes of the declining trogon populations (Table 1).

Most of the trogons and quetzals had incubation periods of 16–19 days (Table 4). The longest incubation period was for the Whitehead's Trogon at 23 days (Table 4). Incubation periods are a function of egg temperatures related to parental incubation attentiveness and evolved intrinsic physiological mechanisms (Martin, Oteyza, Boyce, et al., 2015; Martin, Ton, & Oteyza, 2018). The Whitehead's Trogon had high nest attentiveness (Figure 2a), averaging 91% attentiveness. Such high attentiveness reflects very long on‐bouts by each sex and is typical of many of other members of the trogon family, such as Collared Trogon (Skutch, 1956), Black‐throated Trogon (Skutch, 1959, 1976), Black‐headed Trogon (Skutch, 1948), and Eared Quetzal (Lammertink et al., 1996), although less in Resplendant Quetzal (Skutch, 1944; Wheelwright, 1983) and Elegant Trogon (Hall & Karublan, 1996). The high nest attentiveness, and presumably warm average egg temperatures, together with the long incubation period in the Whitehead's Trogon may enhance immune function of chicks (Arriero et al., 2013; Martin, Arriero, & Majewska, 2011) and aid brain development (Sol et al., 2022). On the other hand, male trogons may not keep eggs as warm as females, such that the high attentiveness by males may not produce high average egg temperatures (Kleindorfer et al., 1995). Study of egg temperature in trogons and physiological development should yield interesting insight.

The long on‐bouts associated with the high attentiveness and low feeding rates of many trogons (see above) can be favored over evolutionary time to reduce parental activity and nest predation risk (Conway & Martin, 2000; Matysioková & Remeš, 2018; Şahin Arslan & Martin, 2024). The latter again suggests the possibility of high nest predation risk over evolutionary time among trogons. If nest predation risk has been consistently high over evolutionary time, their continued existence and diversification across the world suggests their adaptations to nest predation risk have worked over the long term in historic environmental conditions, but possibly not in the currently changing world. The loss and degradation of habitat, with juxtaposition of pastures or agricultural lands may ultimately exacerbate reproductive success and juvenile survival problems.

In summary, the low reproductive success observed in a variety of species and locations may contribute to the widespread population problems among trogons. Our documentation of the poor developmental state at fledging may also indicate an important contribution of juvenile survival to population problems. Detailed studies of these issues in pristine and modified habitats are needed across trogons to better understand causes of their widespread population declines.

## AUTHOR CONTRIBUTIONS

**Necmiye Şahin Arslan:** Conceptualization (equal); formal analysis (equal); writing – original draft (lead); writing – review and editing (equal). **Thomas E. Martin:** Conceptualization (equal); formal analysis (equal); funding acquisition (lead); methodology (lead); writing – original draft (supporting); writing – review and editing (equal).

## CONFLICT OF INTEREST STATEMENT

The authors declare that they have no conflicts of interest.

## Data Availability

Data were deposited in the dryad repository and available for reviewers only at: https://datadryad.org/stash/share/gxsUodxpISzAzvkLCKeXb1hf4U8ruMnFlXtaZ3xdnRw.
